# Cerium Oxide Nanoparticles Achieve Long‐Lasting Senescence Inhibition in an Aging Mouse Model of Sarcopenia via Reactive Oxygen Species Scavenging and CILP2 Downregulation

**DOI:** 10.1002/smsc.202500208

**Published:** 2025-06-26

**Authors:** Wei‐Chih Lien, Yu‐Ling Yu, Ya‐Jyun Liang, Chia‐Yih Wang, Yang‐Chen Lin, Huei‐Cih Chang, Feng‐Huei Lin, Hui‐Min David Wang

**Affiliations:** ^1^ Department of Physical Medicine and Rehabilitation National Cheng Kung University Hospital College of Medicine National Cheng Kung University No. 138, Sheng Li Road Tainan 704 Taiwan (R.O.C.); ^2^ Department of Physical Medicine and Rehabilitation College of Medicine No. 1, University Road Tainan 701 Taiwan (R.O.C.); ^3^ Institute of Biomedical Engineering College of Medicine and College of Engineering National Taiwan University No. 1, Sec. 4, Roosevelt Rd Taipei 106 Taiwan (R.O.C.); ^4^ Department of Cell Biology and Anatomy College of Medicine National Cheng Kung University No. 1, University Road Tainan 701 Taiwan (R.O.C.); ^5^ Institute of Basic Medical Sciences College of Medicine National Cheng Kung University No. 1, University Road Tainan 701 Taiwan (R.O.C.); ^6^ Institute of Biomedical Engineering and Nanomedicine National Health Research Institutes No. 35, Keyan Road, Zhunan Miaoli 350 Taiwan (R.O.C.); ^7^ Graduate Institute of Biomedical Engineering National Chung Hsing University No. 145, Xingda Rd., South Dist. Taichung City 402 Taiwan (R.O.C.); ^8^ Center of Applied Nanomedicine National Cheng Kung University No. 1, University Road Tainan 701 Taiwan (R.O.C.); ^9^ Graduate Institute of Medicine College of Medicine Kaohsiung Medical University No. 100, Shih‐Chuan 1st Road Kaohsiung 807 Taiwan (R.O.C.); ^10^ Department of Medical Laboratory Science and Biotechnology China Medical University No. 91, Hsueh‐Shih Road Taichung City 404 Taiwan (R.O.C.)

**Keywords:** aging, cartilage intermediate layer protein 2, cellular senescence, cerium oxide nanoparticles, reactive oxygen species, sarcopenia

## Abstract

Most drugs used to treat sarcopenia are ineffective. Herein, the long‐acting anti‐sarcopenic properties of cerium oxide nanoparticles (CeNPs) and their underlying mechanisms of action are investigated in aging mice (treated with 4‐hydroperoxy cyclophosphamide (4‐HC)). CeNPs (size, 27.5 nm) with a fluorite crystallization structure are synthesized and subjected to X‐ray diffraction and gas adsorption analyzes. Synthesized CeNPs exhibit Ce^3+^ and Ce^4+^ on their surfaces, a specific surface area within the standard range, and self‐regenerative antioxidative functions. Synthesized CeNPs reduce reactive oxygen species (ROS) levels and exhibit good biocompatibility in muscle satellite (C2C12) cells. According to Rotarod, tensile, and histological analyzes, CeNP treatment once per week in 4‐HC‐treated mice markedly increases muscle strength and the cross‐sectional muscle tissue area relative to that in control mice. Next‐generation sequencing identifies CILP2 as a key differentially upregulated gene common to aging muscle tissues and satellite cells in the presence of ROS. Quantitative polymerase chain reaction and western blotting confirm CILP2, Serpine1, phospho‐p21, Atrogin‐1, and Cxcl10 downregulation in CeNP‐treated mice (compared with 4‐HC‐treated mice); in vitro CILP2 knockdown results in Serpine1 and phospho‐p21 downregulation. These findings confirm the long‐acting effects of CeNPs against sarcopenia in older individuals.

## Graphical abstract

The structure of synthesized cerium oxide nanoparticles (CeNPs) reveals antioxidative properties. CeNP reduces reactive oxygen species and shows good biocompatibility in muscle satellite cells. CeNP‐treated mice exhibit long‐acting anti‐sarcopenic effects through improved muscle mass and strength. CILP2 is identified as a differentially upregulated gene and satellite cell‐linked biomarker. Thus, CeNPs represent an effective treatment for sarcopenia in older individuals.

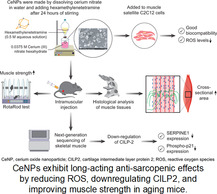

## 1 Introduction

In humans, muscle mass tends to decrease gradually between 30 and 40 years of age, weakening muscle function.^[^ ^1^ ^]^ Between 50 and 60 years of age, muscle mass can decrease by 1%–2% annually; this rate then increases to 3%–5% in older adults,^[^ ^1^ ^]^ resulting in typical muscle mass loss of 30%–50% between 40 and 80 years of age.^[^ ^1^ ^]^ This phenomenon is referred to as sarcopenia and involves a loss of strength, skeletal muscle mass, and muscle integrity that becomes increasingly severe and extensive with age. Sarcopenia is associated with poorer quality of life and physical disability, and in some cases an increased risk of mortality.^[^ ^2^, ^3^ ^]^ The decrease in muscle mass and strength in age‐related sarcopenia occurs more prominently in muscles of the lower limbs than in those of the upper limbs and trunk.^[^ ^4^ ^]^ Considering that the global population aged ≥60 is expected to double between 2015 and 2050, research into anti‐sarcopenia strategies is becoming increasingly crucial.

Nonsteroidal anti‐inflammatory drugs have been successfully used to address various conditions; however, whether short‐term cytokine inhibition prevents sarcopenia or induces adverse effects has not yet been determined.^[^ ^5^ ^]^ Regeneron Pharmaceuticals discovered that combining activin A and myostatin inhibition can improve muscle growth and promote muscle production in mice and monkeys;^[^ ^6^ ^]^ however, results from human studies have yet to be published. Bimagrumab, an experimental drug prepared by Novartis, has been tested on older adults with sarcopenia to evaluate its effects on physical function, skeletal muscle mass, and strength;^[^ ^7^ ^]^ however, no other studies have confirmed its efficacy in treating sarcopenia.

Redox homeostasis can be disrupted by perturbations in pathophysiological conditions during aging. Oxidative or nitrosative stress, which can be induced by H_2_O_2_, reactive oxygen species (ROS), and acrolein, stimulates satellite and muscle cells. Therefore, these cells can be employed as models for investigating in vitro aging‐induced damage in cells such as myoblasts and differentiated myotubes.^[^ ^8^ ^]^ In addition, stress induction alters reconstruction of the extracellular matrix (ECM) in vivo.^[^ ^9^ ^]^ Acrolein is a ubiquitous environmental toxicant that can exert adverse effects on muscle regeneration and mass, leading to conditions such as smoking‐induced sarcopenia.^[^ ^10^ ^]^ Notably, the key regulator of the plasminogen system, Serpine1, also referred to as plasminogen activator inhibitor‐1, worsens sarcopenia.^[^ ^11^ ^]^ Serpine1, which is regulated by redox homeostasis, affects the ECM and alters the behavior of satellite cells and aging muscle.^[^ ^12^ ^]^

Cerium oxide (CeO_2_) nanoparticles (CeNPs) have numerous positive attributes that suggest their potential for managing muscle aging. CeNPs have long‐term effects, are inexpensive, exert few harmful side effects, and exhibit several unique underlying mechanisms. Specifically, CeNPs show self‐regenerative antioxidant potential because of their coexisting oxidation states of Ce^3+^ and Ce^4+^ and the reversible redox switch between these states. A bulk crystal of CeO_2_ primarily comprises Ce^4+^; however, the relative amount of Ce^3+^ increases significantly when the size of CeO_2_ is reduced into nano‐dimensions, which results in enhanced antioxidative effects.^[^ ^13^ ^]^

Existing treatments for sarcopenia involving low‐molecular‐weight antioxidants or antioxidant enzymes have certain limitations, including short half‐lives and a lack of target specificity. Some metal‐based nanomaterials possess an intrinsic antioxidant function similar to that of superoxide dismutase and catalase. These nanomaterials maintain redox homeostasis and modulate immunity.^[^ ^14^ ^]^ Moreover, nanomaterials such as silica NPs and curcumin‐loaded nanolipid carriers promote myoblast differentiation without cytotoxicity in sarcopenia.^[^ ^15^ ^]^ Notably, green nanotechnology has become an emerging field of research, which focuses on developing eco‐friendly products and achieving sustainability. As such, low‐temperature one‐pot syntheses are preferred over traditional pharmaceutical chemistry for metal‐based nanomaterials.^[^ ^16^ ^]^ Eco‐friendly CeNPs demonstrating ROS scavenging abilities have also been synthesized and preclinically evaluated for treating interstitial cystitis,^[^ ^17^ ^]^ chronic pelvic pain syndrome,^[^ ^14^ ^]^ and chronic wounds.^[^ ^18^ ^]^

According to existing literature, most known antioxidants are short‐acting. However, sarcopenia is a chronic condition that requires continued clinical treatment over long periods of time. Therefore, in this study, we examine the long‐acting anti‐sarcopenic properties of CeNPs in aging mice and elucidate the mechanism underlying the actions of upstream Serpine1 regulatory molecules in sarcopenia.

## 2 Results

### 2.1 Characterization of CeNPs

**Figure 1:**
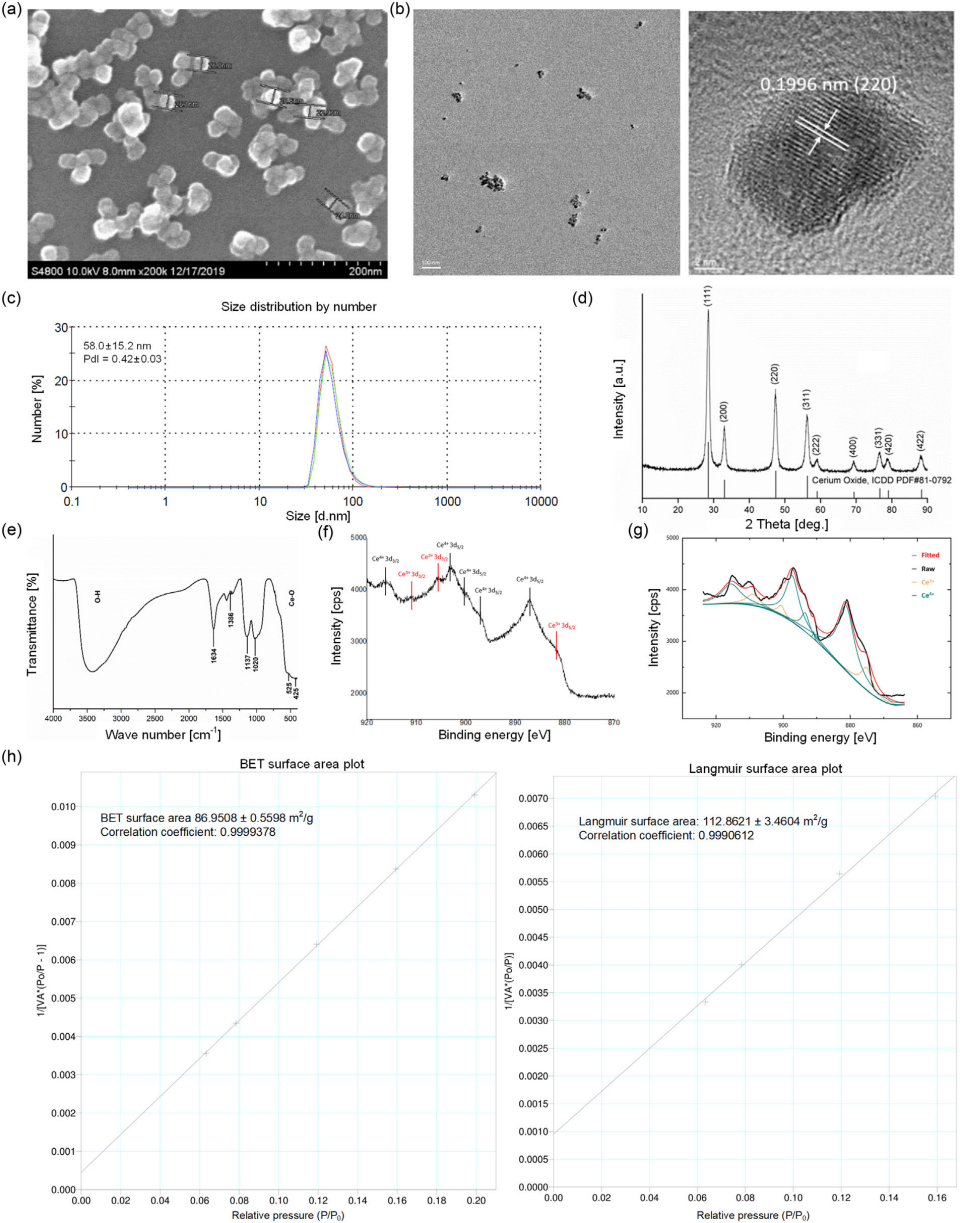
Characterization of CeNPs using various analytical techniques. a) SEM images of CeNPs at a magnification of 200 000 × (scale bar = 200 nm). b) High‐resolution TEM images of CeNPs at a magnification of 30 000 × (scale bar = 100 nm) in the left panel and 800 000 × (scale bar = 2 nm) in the right panel. c) Hydrodynamic size distribution of CeNPs. d) X‐ray diffraction pattern of CeNPs. e) Surface functional group analysis. f) XPS spectrum of CeNPs revealing the binding energy range of cerium in CeNPs, with peaks of Ce^3+^ and Ce^4+^ indicated by red and black lines, respectively. g) Fitted and deconvoluted XPS spectra of CeNPs. h) Specific surface area of individual CeNPs.

The mean diameter of synthesized CeNPs (27.5 nm, n > 100) was measured by scanning electron microscopy (SEM) and Origin software (version 2025), which revealed cube/octahedron‐shaped particles (**Figure** **1**a).^[^ ^19^ ^]^ The larger observed NPs likely formed through the aggregation of smaller particles, which may have occurred because of unstable surface interactions. SEM and transmission electron microscopy (TEM) images revealed a grain size of ≈20 nm (Figure 1a and left panel of Figure 1b). Lattice fringes and d‐spacing were noted in the TEM images, corresponding to the 111 and 200 crystalline planes of standard CeO_2_ (standard data from the Joint Committee on Powder Diffraction Standards 34‐0394; right panel of Figure 1b), respectively. CeNPs in twice‐distilled water had a hydrodynamic size distribution and polydispersity index value of 58.0 ± 15.2 nm and 0.42 ± 0.03, respectively (Figure 1c).

Figure 1d displays the X‐ray diffraction pattern of the CeNPs, revealing distinct peaks related to several crystallographic planes, including (200), (111), (311), (220), (222), (420), (400), (331), and (422). The observed peaks and their intensities aligned with those expected for a cubic fluorite structure (International Center for Diffraction Data [ICDD] powder diffraction file [PDF] #81‐0792), confirming successful CeNP synthesis. The consistency of the peak positions and intensities with standard reference material (ICDD PDF #81‐0792) further confirmed the accuracy of the cubic fluorite structure of synthesized CeNPs. According to the X‐ray diffraction data (Figure 1d), the average CeNP diameter determined by the Scherrer equation was 23.4 nm.

The results of surface functional group analysis for CeNPs are displayed in Figure 1e. The broad absorption band observed between 3200 and 3600 cm^−1^ likely occurred because of O—H stretching vibrations from both adsorbed water molecules and the −OH group on the CeNP surface.^[^ ^20^ ^]^ Conversely, the broad and intense absorption band observed at 425 cm^−1^ was associated with intense Ce—O stretching vibrations.^[^ ^21^ ^]^ The absorption bands at 1634 and 1386 cm^−1^ may be linked to Ce=O vibrations or the bending O—H vibrations of water molecules.^[^ ^21^ ^]^ These findings align with those of previous literature and indicate successful CeNP synthesis.

The results of X‐ray photoelectron spectroscopy (XPS) analysis presented in Figure 1f indicate the presence of 3D‐orbit photoelectron peaks corresponding to trivalent and tetravalent cerium ions on the surface of the synthesized CeNPs. These ions exhibit antioxidant properties.^[^ ^14^ ^]^ XPS also revealed chemical bonding peaks that were ascribed to Ce^3+^ (red arrows in Figure 1h) and Ce^4+^ (black arrows in Figure 1f) at the corresponding binding energies. We identified peaks at 875–895 and 895–910 eV, which were ascribed to Ce 3d_5/2_ and Ce 3d_3/2_ levels of degradation, respectively. Deconvoluted peaks were identified at 875.100, 900.650, and 909.000 eV, which were attributed to Ce^3+^ oxidation states. Deconvoluted peaks were also noted at 881.240, 890.900, 893.400, 897.300, and 915.550 eV, which were ascribed to the Ce^4+^ state of cerium ions (Figure 1f). Fitting and deconvolution of the XPS spectra revealed a Ce^4+^/Ce^3+^ ratio of 0.963 for the CeNPs (Figure 1g).

Figure 1h illustrates the specific surface area of CeNPs, determined using a specific surface area and porosity analyzer. Each CeNP had a Brunauer–Emmett–Teller surface area of 86.9508 ± 0.5598 m^2^/g and a Langmuir surface area of 112.8621 ± 3.4604 m^2^/g. These values fall within the expected or normal ranges for such measurements.^[^ ^22^ ^]^ A methylene blue assay was performed to determine the antioxidant capacity of the CeNPs, in which methylene blue changes color from blue (oxidized form) to colorless (reduced form) when reduced by an antioxidant. The absorbance value (indicating the total concentration of the oxidized form of methylene blue) of the control group was higher than that of the CeNP group after 1 min. Figure S1, Supporting Information, depicts changes in the absorbance value during a 0–4 min reaction with H_2_O_2_ in the CeNP and control groups. The total concentration of the oxidized form increased as the reaction time increased. In the CeNP group, H_2_O_2_ primarily oxidized the CeNPs, which delayed the conversion of colorless methylene blue to its blue form. Owing to their ability to scavenge ROS, CeNPs are considered high‐level antioxidants. Conversely, in the control group without CeNPs, H_2_O_2_ mainly oxidized the colorless methylene blue, increasing the absorbance values. After 1 to 2 min, the reducing capacity of the CeNPs was exhausted, allowing the oxidation effect of H_2_O_2_ to resume. As a result, we observed no significant difference in the concentration of methylene blue between the CeNP and control groups at 2–4 min (Figure S1, Supporting Information). The XPS, specific surface area, and methylene blue assay results indicate that the synthesized CeNPs possess notable catalytic and antioxidant properties, highlighting their potential for applications requiring these properties.^[^ ^14^, ^22^ ^]^

### 2.2 Influence of 4‐HC on C2C12 Cell Viability, Rescue of 4‐HC‐induced Loss of Cell Viability by CeNPs, Intracellular ROS Measurement, and RNA Sequencing Validation

**Figure 2:**
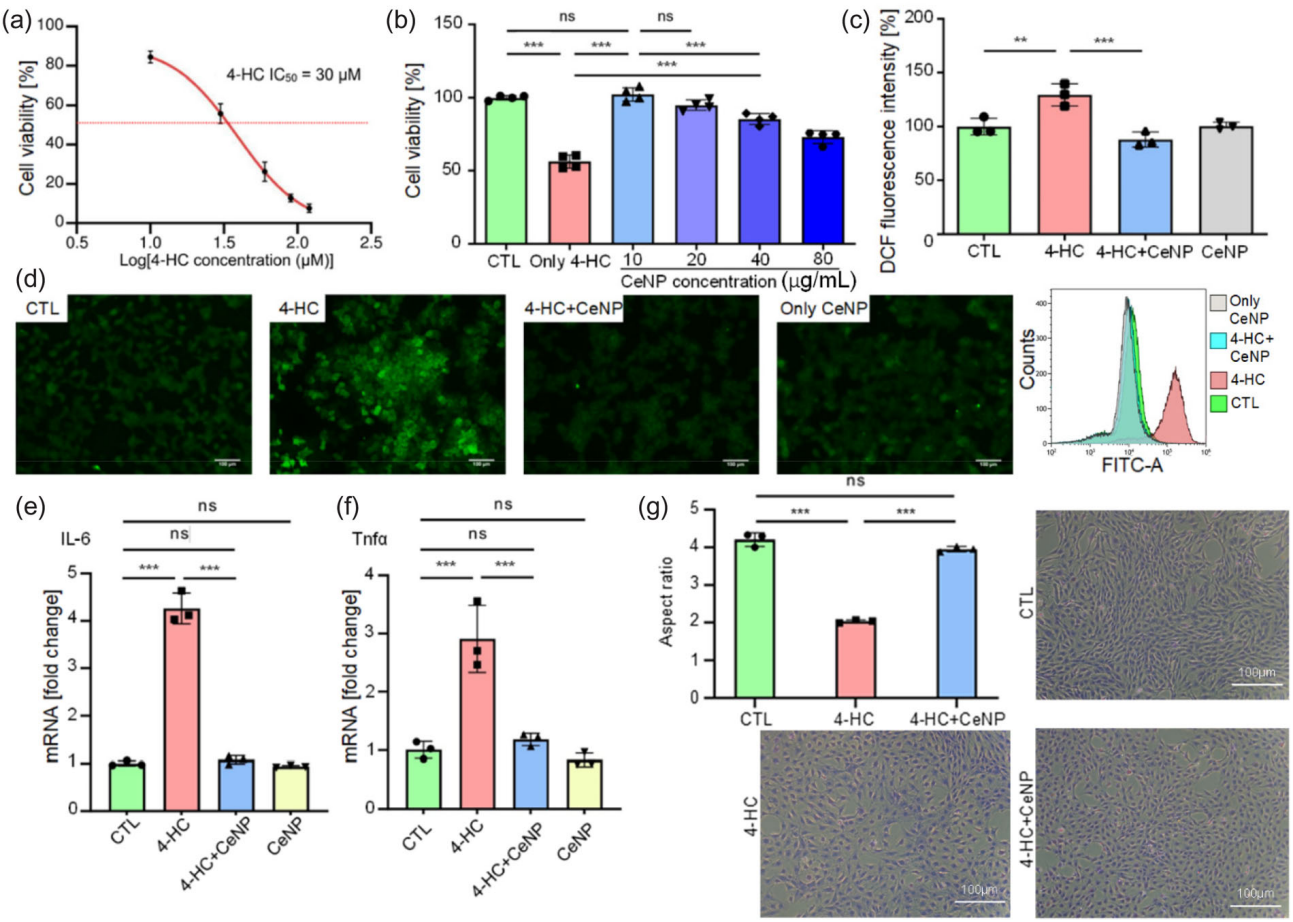
Impact of 4‐hydroperoxy cyclophosphamide (4‐HC) on C2C12 cell viability, rescue of 4‐HC‐induced cell loss through CeNP treatment, and measurement of intracellular ROS level and pro‐inflammatory cytokine expression. a) C2C12 cell viability decreased with exposure to varying concentrations of 4‐HC, with particularly notable decreases at ≥30 μM; thus, 30 μM was considered the half‐maximal inhibitory concentration (n = 4). b) Application of CeNPs for 24 h at 10, 20, 40, and 80 μg/mL prevented the loss of 4‐HC‐induced C2C12 cell viability (n = 4). c) 2′,7′‐dichlorofluorescin diacetate (DCFDA) assay revealed intracellular ROS levels in different cell groups, with CeNP treatment exhibiting a positive correlation with reduced ROS content (n = 3). d) Corresponding DCFDA assay images (left panel) and flow cytometric histograms showing dichlorofluorescein (DCF) fluorescence (FITC‐A) in C2C12 cells (n = 3 each) (right panel). e) and f) CeNP demonstrated anti‐inflammatory effects on 4‐HC‐treated C2C12 cells, as evidenced by downregulation of (e) Il6 and (f) Tnfα expression in the CeNP + 4‐HC group relative to that in the 4‐HC group (n = 3). g) Cell elongation and aspect ratios in control, 4‐HC, and 4‐HC + CeNP groups (n = 3). Values are the mean ± SD. Data were compared via one‐way ANOVA with Bonferroni correction. *p < 0.05; **p < 0.01; and ***p < 0.001; ns, not significant.

Within aqueous solutions, 4‐hydroperoxy cyclophosphamide (4‐HC) undergoes rapid hydrolysis, forming 4‐hydroxycyclophosphamide, which spontaneously converts into acrolein (a major toxicant in cigarette smoke) and induces skeletal muscle catabolism.^[^ ^14^ ^]^ Here, treating C2C12 cells with 30 μM of 4‐HC led to 50% lower cell viability (measured as IC_50_). Therefore, we employed this concentration to reduce cell viability in subsequent experiments (**Figure** **2**a). We tested various CeNP concentrations before 4‐HC induction and noted the prevention of 4‐HC‐induced cytotoxicity using CeNPs. Further experiments revealed that 10 μg/mL of CeNPs effectively mitigated the ability of 4‐HC to reduce cell viability (Figure 2b). In addition, as indicated by the level of green fluorescence in the 2′,7′‐dichlorofluorescein diacetate (DCFDA) assay, 30 μM of 4‐HC significantly triggered ROS production in C2C12 cells (Figure 2c). To confirm that CeNPs inhibit the ability of 4‐HC to induce cellular oxidative stress, we analyzed C2C12 cells under the following four conditions: control, exposure for 4 h to 30 μM of 4‐HC to induce oxidative stress, pretreatment for 24 h with 10 μg/mL of CeNPs followed by 4 h of 30 μM 4‐HC induction, and treatment for 24 h with 10 μg/mL of CeNPs. The results revealed that CeNPs inhibited the cellular oxidative stress response of 4‐HC (Figure 2c,d). The DCFDA assay demonstrated the ability of CeNPs to counteract 4‐HC‐induced oxidative stress in vitro. Subsequently, RNA extraction was performed on the four cell groups for quantitative polymerase chain reaction (qPCR) analysis. The qPCR results for Tnfα and Il6 indicated that CeNPs can downregulate these pro‐inflammatory cytokines induced by 4‐HC in vitro (Figure 2e,f). Additionally, cell elongation analysis revealed a higher aspect ratio in the control and 4‐HC + CeNP groups relative to that in the 4‐HC group (Figure 2g).

**Figure 3:**
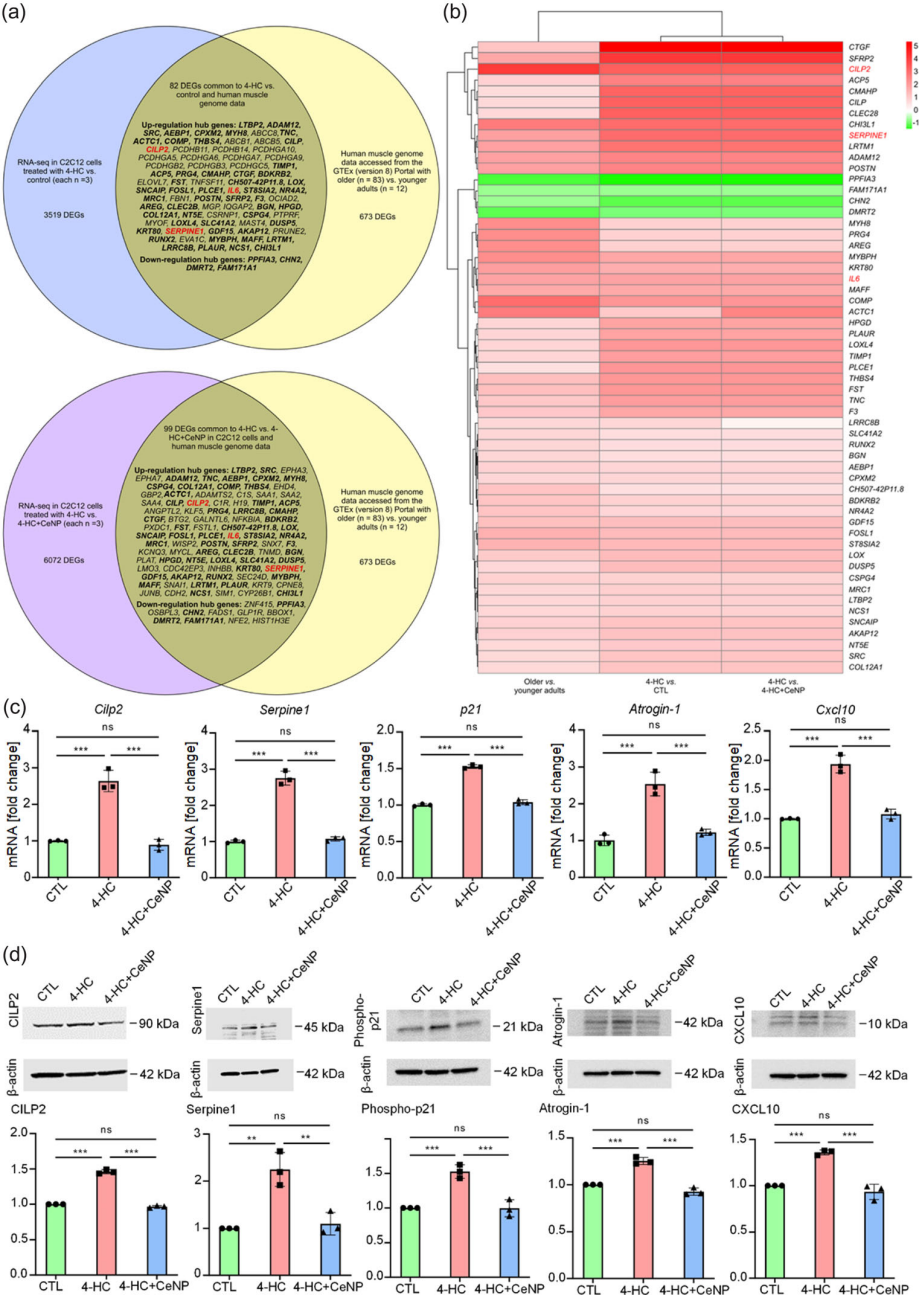
RNA sequencing analysis comparing DEGs in human muscle data accessed from the GTEx (version 8) Portal for older (n = 83) versus younger adults (n = 12) and C2C12 cells, validated using qPCR, western blot analysis, and siRNA knockdown in C2C12 cells. a) The 82 DEGs common to 4‐HC versus control (CTL) in C2C12 cells and human muscle genome data (older adults vs. younger adults) are shown in the upper panel. The 99 DEGs common to 4‐HC versus 4‐HC + CeNP in C2C12 cells and human muscle genome data (older adults vs. younger adults) are shown in the lower panel. The 57 DEGs common to both panels are shown in bold. The expression of inflammatory response genes, such as Il6 and Serpine1 (in red bold text), as well as the ECM gene CILP2 (in red bold text), were more significantly upregulated in older adult muscle genome data relative to younger adult muscle genome data and in the 4‐HC group relative to the control and 4‐HC + CeNP groups. b) Corresponding heatmap of the 57 DEGs common to 4‐HC versus CTL and 4‐HC versus 4‐HC + CeNP in C2C12 cells and human muscle genome data (older adults vs. younger adults). CILP2 (in red text) was the top upregulated DEG in the left column (human muscle genome data). c) qPCR results for different C2C12 cell groups (n = 3 in each group). Expression of CILP2, Serpine1, p21, Atrogin‐1, and Cxcl10 in C2C12 cells. d) Western blot analysis of different C2C12 cell groups (n = 3 in each group). Expression of CILP2, SERPINE1, phospho‐p21, Atrogin‐1, and CXCL10 in C2C12 cells. Values are the mean ± SD. Data were compared via one‐way ANOVA with Bonferroni correction. *p < 0.05; **p < 0.01; ***p < 0.001; ns, not significant.

RNA sequencing analysis of muscle genome data for older adults and 4‐HC‐treated C2C12 cells in vitro revealed differentially expressed genes (DEGs) relative to muscle genome data for younger adults and control and CeNP‐treated C2C12 cells, respectively. A comparison of heatmaps between older adult versus younger adult groups, 4‐HC versus control, and 4‐HC versus 4‐HC + CeNP indicated that the expression of inflammatory response genes, such as Il6, Serpine1, and the ECM gene CILP2, was more significantly upregulated in the older adult group relative to the younger adult group and in the 4‐HC group relative to the control and 4‐HC + CeNP groups (**Figure** **3**a,b). The derived gene ontology (GO) enrichment analysis results are displayed in Figure S2, Supporting Information. Protein–protein interactions with an interaction score of >0.15 for DEGs were obtained from the STRING database; CILP2 and SERPINE1 were identified as interacting proteins (Figure S3, Supporting Information). Among the DEGs, CILP2 was ranked highly in the older adult group relative to the younger adult group in 4‐HC relative to the control group, and in 4‐HC relative to the 4‐HC + CeNP group (Figure 3b), and was identified as a hub gene (Figure S3, Supporting Information). RNA sequencing data were validated using qPCR (Figure 3c) and western blot analysis (Figure 3d). The qPCR results revealed increased levels of *CILP2,* Serpine1, p21, Atrogin‐1, and Cxcl10 in the 4‐HC group relative to the control and 4‐HC + CeNP groups (n = 3 per group; Figure 3c). Western blot results indicated elevated levels of CILP2, SERPINE1, phospho‐p21, Atrogin‐1, and CXCL10 within the 4‐HC group relative to the control and 4‐HC + CeNP groups (n = 3 per group; Figure 3d). CILP2 was knocked down in C2C12 cells (n = 3 per group; Figure S4, Supporting Information). CILP2 knockdown in C2C12 cells treated with 4‐HC led to the downregulation of SERPINE1 (associated with the inflammatory response) and phospho‐p21 (associated with cellular senescence), as indicated in Figure S4, Supporting Information (n = 3 per group).

### 2.3 Behavior, Serum, and Histology of Aging Mouse Muscles and Effects of CeNP Treatment

**Figure 4:**
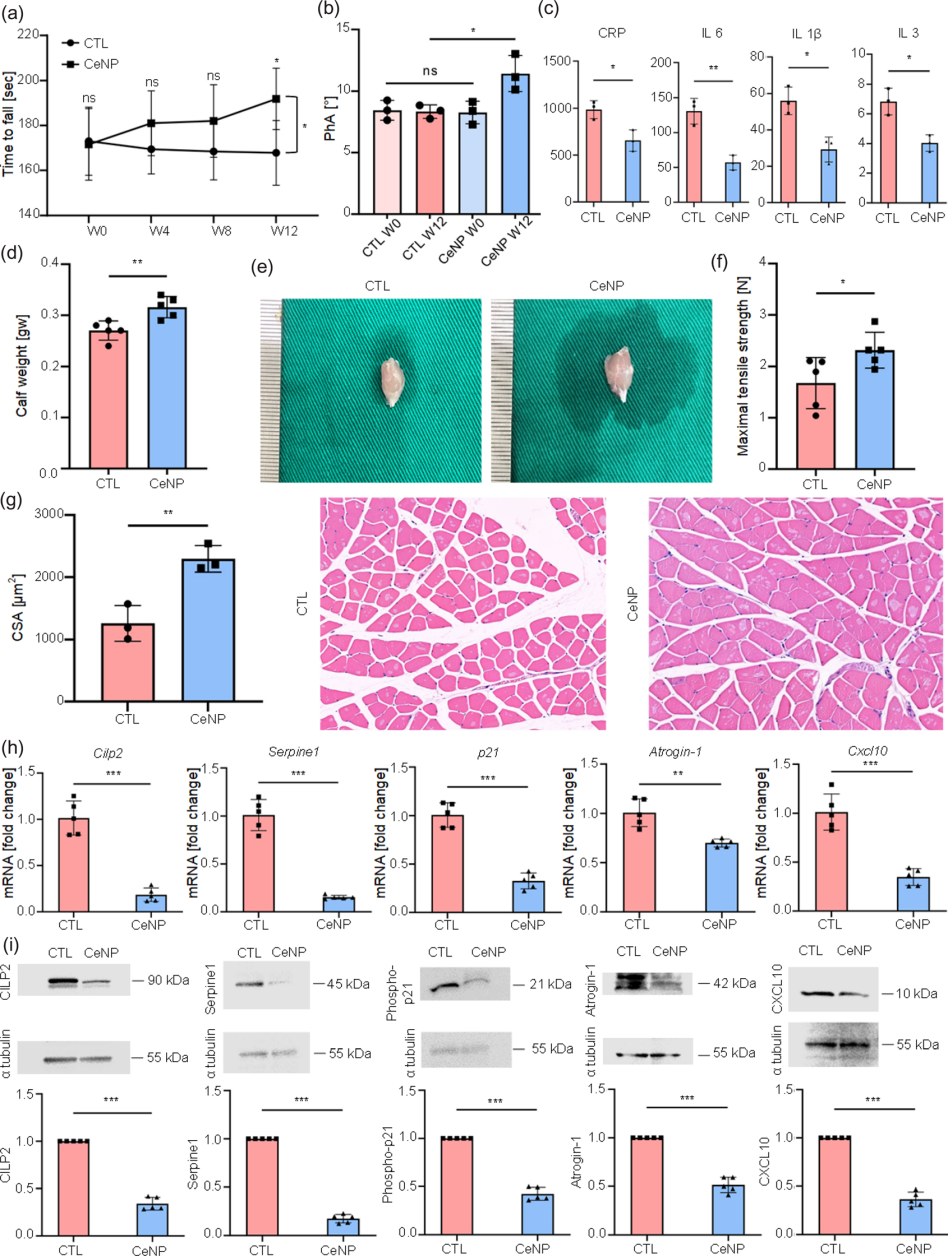
Comparison of the behavioral, biomechanical, histological, and biochemical characteristics of aging C57/BL6J mice after 12 weeks of CeNP treatment relative to the control (CTL) group. a) Rotarod running test in aging C57/BL6J mice (n = 5 each). b) Bioelectrical impedance analysis, including phase angle (PhA) calculation (n = 3 each). c) Serum C‐reactive protein (CRP), IL‐1β, IL‐3, and IL‐6 levels in aging C57/BL6J mice in the CTL and CeNP‐treated (CeNP) groups. d) Calf weight (n = 5 each). e) Corresponding images of the calf muscle of aging C57/BL6J mice. f) Ex vivo analysis of muscle mechanical properties (maximum tensile strength [Newton, N] during elongation at failure) determined by tensile tests (n = 5 each). g) Histological examination of CTL and CeNP groups with quantification of the CSA in CeNP versus CTL groups (each n = 3) and corresponding images of quadriceps muscle histology. h) qPCR results in aging C57/BL6J mice in CTL and CeNP groups. Expression of CILP2, Serpine1, p21, Atrogin‐1, and Cxcl10 in CTL and CeNP groups (n = 5 each). i) Western blot analysis of aging C57/BL6J mice in CTL and CeNP groups. Expression of CILP2, SERPINE1, phospho‐p21, Atrogin‐1, and CXCL10 in CTL and CeNP groups (n = 5 each). Values are the mean ± SD. Data were compared via a two‐way repeated measurement ANOVA with Dunnett's post‐hoc test (a) and unpaired two‐tailed *t*‐tests (b‐i). *p < 0.05; **p < 0.01; ***p < 0.001; ns, not significant.

The Rotarod test revealed that 12 weeks of CeNP treatment improved the behavioral performance of mice from that in the control group (**Figure** **4**a). The CeNP group demonstrated an increased phase angle (Figure 4b), serum C‐reactive protein and pro‐inflammatory cytokine levels (IL‐6, IL‐1β, and IL‐3) (Figure 4c), and calf weight relative to the control group (Figure 4d,e). Serum levels of creatine kinase, lactate dehydrogenase, alanine aminotransferase, and creatinine in the control and CeNP groups showed no differences and were within the normal ranges for liver and kidney function (Table S1).^[^ ^23^ ^]^ Regarding the ex vivo muscle mechanical properties of mice assessed using tensile testing, the CeNP group exhibited higher maximum tensile strength (Newton, N) during elongation at failure than the control group (Figure 4f). Histological examination of quadriceps muscles revealed a larger cross‐sectional area (CSA) in the CeNP group than in the control group (Figure 4g). qPCR analysis (Figure 4h) indicated downregulated expression of CILP2, Serpine1, p21, Atrogin‐1, and Cxcl10 in the CeNP group relative to that in the control group, which was subsequently validated by western blot analysis (Figure 4i). TEM images revealed the presence of tubular aggregates^[^ ^24^ ^]^ in muscle samples from the control group and CeNPs in the cytoplasm of muscle and liver cell samples from the CeNP group (Figure S5, Supporting Information).

## 3 Discussion

To the best of our knowledge, this study represents the first comprehensive in vitro and in vivo evaluation of the inhibitory effect of CeNPs on senescence in sarcopenia. Older individuals with sarcopenia face an increased risk of osteoporosis, falls, hospitalization, institutionalization, emergency department visits, and mortality. However, our understanding of the pathogenesis and progression of sarcopenia remains limited. Our findings indicate that CeNPs exhibit considerable biocompatibility, as evidenced by their nontoxic effects on C2C12 cells and the lack of significant adverse reactions in aging mice. Our findings also suggest that CeNPs may exert an immunomodulatory effect in sarcopenia through their ROS scavenging ability and downregulation of CILP2 in muscle tissue. CILP2 downregulation may be associated with reduced SERPINE1 and phospho‐p21 expression in C2C12 cells. Accordingly, our results indicate that CeNP therapy holds potential as a long‐acting therapeutic strategy for sarcopenia in older individuals.

Cellular senescence is crucial in various biological processes because it contributes to skeletal muscle wasting, weakness, and other age‐related disorders.^[^ ^25^ ^]^ Therefore, cellular senescence is a potential therapeutic target for such conditions. Delaying the onset of sarcopenia, which involves a decrease in muscle mass and strength, may be achievable by modulating fundamental aging mechanisms, including cellular senescence. CeNPs have previously been applied to proliferating C2C12 mouse skeletal muscle cells under gravitational unloading and cosmic radiation, and to murine muscle cells and human skeletal myoblasts for the in vivo treatment of muscle wasting induced by spaceflight in low Earth orbit and deep space.^[^ ^26^, ^27^, ^28^ ^]^ The in vitro results confirmed that CeNPs exhibit excellent antioxidant and anti‐inflammatory activities.^[^ ^29^, ^30^ ^]^ Moreover, evidence from preclinical models has revealed promising interventions that may reduce cellular senescence and its associated consequences, including senescent cell accumulation, chronic inflammation, and oxidative stress,^[^ ^31^ ^]^ thereby combating age‐related sarcopenia.

A key concern related to muscle aging and sarcopenia development is the dysfunction of satellite cells. SERPINE1 is a mediator and marker of cellular senescence,^[^ ^32^ ^]^ and p21 induces a senescence program and skeletal muscle dysfunction associated with SERPINE1.^[^ ^33^ ^]^ Our study revealed that the in vitro treatment of C2C12 cells with 4‐HC can significantly upregulate pro‐inflammatory cytokines, including IL‐6, TNF*α*, and CXCL10, and increase the expression of Atrogin‐1, SERPINE1, and p21 (Figure 2, 3), causing C2C12 cell elongation. However, after treatment with CeNP, these pro‐inflammatory cytokines and cellular senescence markers were downregulated. In male C57/BL6 mice, serum IL‐6 levels increase significantly after 12 months of age following a decline in hindlimb muscle mass.^[^ ^34^ ^]^ In our aging mouse model, CeNP treatment exhibited anti‐sarcopenic effects and reduced pro‐inflammatory cytokine expression in both the muscles and serum (Figure 4).

Age‐related factors such as skeletal muscle satellite cell dysfunction, immunosenescence, and disruptions in redox homeostasis^[^ ^13^ ^]^ can deteriorate skeletal muscle structure and function, resulting in sarcopenia. Oxidative stress plays a critical role in regulating the immune response and initiating inflammatory responses in the body.^[^ ^13^ ^]^ Notably, inflammation, particularly when uncontrolled and self‐sustaining, can lead to various diseases, with inflammatory mediators, signaling molecules, and ROS all linked to chronic disease.^[^ ^35^ ^]^ Inorganic nanotechnology‐based antioxidants have gained increasing attention for their potential to treat diseases caused by excessive ROS production because their synthesis reaction can be controlled, and they exhibit excellent catalytic efficiency, stability, and biocompatibility.^[^ ^14^ ^]^ Cocteau et al. employed a mouse model to show that Cans exhibit catalase and oxidase activity and effectively improve muscle strength and longevity in mice with amyotrophic lateral sclerosis.^[^ ^36^ ^]^ In our study, Cans showed the potential to scavenge ROS by directly interacting with them or mimicking natural antioxidant enzymes (Figure 2).

Rejuvenating aged muscles through anti‐senescence treatments is generally considered advantageous for older adults with sarcopenia. Declining muscle function may be linked to muscle mass reduction and qualitative muscle changes.^[^ ^37^ ^]^ In this study, we demonstrated that CeNP treatment enhanced muscle function by increasing muscle mass, improving muscle quality, and exerting anti‐senescent effects by modulating senescence biomarkers such as SERPINE1 and p21 (Figure 3 and 4). Lifestyle modifications, including smoking cessation to reduce the production of harmful metabolites such as acrolein,^[^ ^10^ ^]^ and pharmacotherapies with anti‐inflammatory properties may therefore offer benefits for the treatment of sarcopenia. Elevated systemic levels of pro‐inflammatory cytokines, including IL‐6, SERPINE1, and TNFα, may contribute to age‐related muscle decline.^[^ ^5^ ^]^ SERPINE1 in fibrotic lungs induces cell senescence by activating p21.^[^ ^38^ ^]^ Although aging satellite cells exhibit heightened susceptibility to apoptosis related to p21 activation, the downregulation of p21 in these cells can notably enhance muscle mass and function in adult mice.^[^ ^39^ ^]^

The plasminogen system is a crucial proteolytic system in ECM remodeling. SERPINE1 is a crucial regulator of this system that exacerbates various diseases by promoting ECM accumulation and altering cell behavior.^[^ ^12^ ^]^ SERPINE1 has also been linked to regeneration and pathologies in skeletal muscle, although its complete function in muscle remains unclear.^[^ ^12^ ^]^ Thus, understanding SERPINE1 changes in skeletal muscle is essential because this organ plays a crucial role in overall health and quality of life.

CILP2 is an ECM‐related protein identified as a potential biomarker for spinal muscular atrophy^[^ ^40^ ^]^ and knee osteoarthritis.^[^ ^41^ ^]^ CILP2 is also present in skeletal muscle and may influence the structure and function of non‐cartilaginous tissues.^[^ ^39^ ^]^ CILP2 is reportedly associated with insulin resistance^[^ ^42^ ^]^ and may play a role in ECM protein interactions related to myofibrosis in dystrophinopathy.^[^ ^43^ ^]^ Impaired function of satellite cells in muscle with age generally results from intrinsic changes in these cells and alterations in local and systemic environments in the form of, for example, changes in myofibers, fibro‐adipogenic progenitors, ECM, and signaling cues from the immune system. According to our results, including the GO enrichment results of pathways involved in the ECM and collagen fiber organization in 4‐HC‐treated C2C12 cells relative to the control (Figure S2, Supporting Information), disruption of satellite cell functioning in muscle regeneration with aging^[^ ^44^ ^]^ may be reversed by CeNP treatment through the downregulation of CILP2, SERPINE1, and p21 (Figure 3).

Nevertheless, this study had some limitations. First, we evaluated only aging male C57BL6/J mice treated with CeNPs for 12 weeks; therefore, future research should investigate the therapeutic effects of CeNP treatment in female mouse models with accelerated aging. Second, the precise mechanism through which CILP2 knockdown downregulates SERPINE1 and phospho‐p21 remains unclear. Thus, further research is required to elucidate the signaling pathways influencing CILP2 and understand the role of pro‐inflammatory chemokines and biomarkers of cellular senescence in both human and mouse models of sarcopenia.

## 4 Conclusion

We synthesized CeNPs that exhibit considerable biocompatibility and anti‐senescent effects in sarcopenia through their ability to scavenge ROS and downregulate CILP2 in muscle tissues, which may reduce SERPINE1 and phospho‐p21 expression. Thus, CeNPs represent an effective treatment for sarcopenia in older individuals because of their long‐term anti‐sarcopenic effects. Our aging mouse model and CeNP treatment protocol can be employed in future studies investigating the pathogenesis of sarcopenia and the effects of CeNP treatment. Moreover, our findings can be translated to larger animal models and human patients to expand the research applications.

## 5 Experimental Section

5.1

5.1.1

##### CeNP Production

We employed an altered version of the environmentally friendly method presented by Shah et al. for green nanotechnology.^[^ ^16^, ^17^ ^]^ CeO_2_ powders were acquired by adding NO_3_^−^ ligands to a solution of cerium ions, i.e., cerium (III) nitrate hexahydrate,^[^ ^45^ ^]^ obtained from Alfa Aesar (99.5%; Waltham, MA, USA). In brief, CeNPs were prepared by dissolving 0.0375 M of cerium (III) nitrate hexahydrate in 50 mL of water, twice subjected to distillation at 24 ± 2 °C. The sample was stirred for 24 h at the same temperature, and 50 mL of 0.5 M aqueous solution of hexamethylenetetramine (99.5%; Alfa Aesar) was added. The solubility point was surpassed to obtain a precipitate from this reaction. The results suggest that an increase in temperature changed the morphology of the NPs from cubic to hexagonal, whereas a decrease in the temperature of the reaction atmosphere decreased the size of the NPs.^[^ ^46^ ^]^ We subjected the solution to 30 min of centrifugation at 9961 × g to separate CeNPs and enable the collection of CeNP sediment. We then used 95% ethanol (Alfa Aesar) and deionized water to wash the sediments twice in preparation for subsequent SEM measurements. Next, we resuspended the CeNPs in phosphate‐buffered saline (PBS) with a pH of 7.4, then subjected the particles to 15 min of autoclave sterilization at 121 °C in preparation for subsequent in vitro and in vivo analyzes.

##### CeNP Characterization

We used an SEM device (Hitachi S‐4800 Field Emission SEM device, Tokyo, Japan), operated at a voltage of 10 kV, to analyze the size and shape of CeNPs. The collected particles were carefully fixed on a copper stub and then subjected to gold sputtering. The Nano Measurer program package (version 1.2) established by Jie Xu at Fudan University in China, was used to analyze the SEM images. Images of >100 CeNPs were examined at a magnification of 200 000 × to determine their average size using Origin software (version 2025).^[^ ^19^ ^]^ The CeNPs were also analyzed using TEM, and their hydrodynamic size, surface area, X‐ray diffraction pattern, and surface characteristics were measured. The antioxidant activity of CeNP was analyzed using a methylene blue assay. Details of these analyzes are presented in the Supporting Information.

##### C2C12 Cell Culture and in Vitro Analysis of Smoking‐Induced Sarcopenia

C2C12 cells (BCRC No. 60 083, Bioresource Collection and Research Center, Hsinchu, Taiwan) represent an immortal mouse cell line of skeletal myoblasts originally derived from adult mouse satellite cells. To culture these cells, we used 90% McCoy's 5 A medium from Sigma‐Aldrich (St. Louis, MO, USA) along with 1% v/v penicillin/streptomycin, 10% v/v fetal bovine serum, and 1.5 mM L‐glutamine. We incubated the cells under 5% CO_2_ in humid air at 37 °C. The cells were then treated with 4‐HC in vitro to reduce viability.^[^ ^14^ ^]^ The water‐soluble tetrazolium salt‐1 (WST‐1) assay (MK400, Takara, Shiga, Japan) was used according to the manufacturer's instructions to assess cell viability and determine the impact of 4‐HC and CeNP treatment on cell survival. We placed the C2C12 cells in tissue culture plates with 96 wells at a cell seeding density of 10 000 cells per cm^2^. Next, the cells were incubated for 24 h until full cell adhesion was achieved, followed by 4 h of exposure to varying concentrations of 4‐HC (0, 10, 30, 60, 90, and 120 μM). PBS was used to wash the cells before introducing them into the culture medium. After culturing 100 μL cells per well, we added a 10 μL PerMix WST‐1 solution (resulting in final dilution of 1:10). The cells were then incubated for 2 h in darkness under 5% CO_2_ at 37 °C. The optical density of the formazan product was measured at 450 nm with a Tecan Sunrise Plate Reader (Männedorf, Switzerland) using a colorimetric detection method (n = 4). We determined the cell viability percentage according to Equation (1):

(1) $$Cell\;viability\left(\%\right)=\left[\frac{\left(OD_{sample}-OD_{blank}\right)}{\left(OD_{control}-OD_{blank}\right)}\right]\times 100$$

##### Effective Concentration and Therapeutic Effects of CeNPs by In Vitro Analysis of Smoking‐Induced Sarcopenia

We determined the effective concentration of CeNPs by treating C2C12 cells with CeNP concentrations of 10, 20, 40, and 80 μg/mL at full adhesion for 24 h. We added the half‐maximal inhibitory concentration (IC_50_) of 4‐HC to the culture medium and allowed it to stand for 4 h to induce cell damage. The WST‐1 assay was performed on the following four groups of cells: negative control, positive control treated with 4‐HC, 4‐HC with 10 μg/mL CeNPs, and 4‐HC with 20 μg/mL CeNPs (n = 4 in each group).

To evaluate the scavenging effects of CeNP treatment on intracellular ROS, and corresponding change in mRNA and cell morphology, we divided C2C12 cells into four groups: a positive control group that received 4‐HC treatment; a negative control group; a group exposed to 10 μg/mL CeNPs for 24 h; and a group exposed to 10 μg/mL CeNPs for 24 h before being induced for 4 h with 30 μM of 4‐HC. The procedures for detecting intracellular ROS using the DCFDA assay are described in the Supporting Information (n = 3 in each group). Flow cytometry (CytoFLEX, Beckman Coulter, Brea, CA, USA) analysis of DCFDA assays was performed as described in the Supporting Methods.

C2C12 cells were treated with TRIzol reagent obtained from Invitrogen (Carlsbad, CA, USA) for total RNA extraction. In addition, Bioline's SensiFAST cDNA Synthesis kit (London, UK) was used to synthesize cDNA with random hexamers and oligo (dT) primers. We used a qPCR system (StepOne Software version 2.2) from Thermo Fisher Scientific (Waltham, MA, USA)^[^ ^14^ ^]^ to evaluate each sample three times for specific genes (cartilage intermediate layer protein 2 [CILP2], Serpine1, p21, Atrogin‐1, and Cxcl10) and the endogenous control, α‐tubulin. We obtained triplicate measurements of the cycle threshold (Ct) and used the means of these measurements in subsequent analyzes. We adjusted the Ct value of each gene to α‐tubulin to calculate the relative gene expression as per the 2^−ΔΔCt^ method. Table S2, Supporting Information lists the primer sequences, and the Supporting Methods detail the RNA sequencing methods employed in this study.

The cellular proteins associated with Atrogin‐1 for muscle catabolism, phospho‐p21 for senescence, Serpine‐1 and Cxcl10 for inflammatory response, and CILP2, which were identified from next‐generation sequencing analysis, were quantified using western blotting. We incubated the blots with primary antibodies, including anti‐CILP2 (11 813‐1‐AP, Proteintech, Rosemont, IL, USA; 90 kDa), anti‐Serpine1 (66 261‐1‐Ig, Proteintech, Rosemont, IL, USA; 45 kDa), anti‐p21 (phospho‐T145; A27422, Antibodies‐online, Aachen, Germany; 21 kDa), anti‐Atrogin‐1 (67 172‐1‐Ig; Proteintech, Rosemont, IL, USA; 42 kDa), anti‐Cxcl10 (#ab9938, Abcam, Cambridge, MA, USA; 10 kDa), and anti‐*β*‐actin (GTX26276; GeneTex, Irvine, CA, USA; 42 kDa), for 16 h at 1:7000 dilution each at 4 °C. Subsequently, we incubated the blots with the relevant secondary antibodies for 1 h at 37 °C. Finally, we visualized the protein bands with the Hansor Luminescence Image System (M3‐8068; Taichung, Taiwan).^[^ ^14^ ^]^

##### C2C12 Cell Elongation

C2C12 cells were seeded in culture plates with 12 wells at a cell density of 10 000 cells per cm^2^. When the seeded cells reached ≈70% confluence, the medium was substituted with Dulbecco's modified Eagle medium (Gibco) enriched with 2% v/v horse serum, 100 units mL^−1^ v/v penicillin/streptomycin, and 2 mM glutamine (Gibco).^[^ ^47^ ^]^ After incubating the cells in a differentiation medium for 72 h, we performed the same treatment procedures as those for CeNPs and 4‐HC, with the cells divided into the same treatment groups. The medium was replaced every 24 h. Following treatment with pre‐cooled methanol for 30 s, the cells were stained for 10 min using a 0.1% crystal violet solution, then rinsed with distilled water and subjected to microscopic imaging. We conducted morphological analysis by evaluating cell elongation at 20 × magnification. We then employed ImageJ software (version 1.53, National Institutes of Health, USA) to quantify the aspect ratio, derived by dividing the lengths of the longest lines across the nuclei by those of the shortest. An average measurement was obtained from ≈100 cells assessed in five randomly selected fields. Data were collected over three independent experiments.

##### Human Genome Data

Human skeletal muscle data were accessed from the GTEx (version 8) Portal, as previously described.^[^ ^48^ ^]^ The raw counts and normalized transcripts per million matrices for RNA sequencing were obtained directly from the Portal (https://gtexportal.org/home/datasets) in the GENCODE (https://www.gencodegenes.org/) project (release 26). To analyze muscle aging in deceased male donors whose deaths were classified on the Hardy scale as either “1” (indicating a violent and fast death) or “2” (indicating a fast death of natural causes), we obtained 153 RNA sequencing samples from the gastrocnemius muscles of individuals aged between 20 and 79. Patients aged 40–59 (n = 55) were excluded because sarcopenia is generally detected in patients aged ≥60.^[^ ^1^ ^]^ The remaining GTEx skeletal muscle data were divided into young to middle‐aged (younger adults aged 20–39, n = 12) and old‐aged (older adults aged 60–79, n = 86) groups for further analysis.

We employed the fastp program (version 0.20.0) to identify low‐quality bases and sequences from adapters in the raw data, which were subsequently excluded.^[^ ^49^ ^]^ We employed HISAT2 (version 2.1.0) to align the retained reads to the reference genomes.^[^ ^50^ ^]^ Gene abundance was quantified using FeatureCounts software (v2.0.1) with the Subread package.^[^ ^51^ ^]^ We identified DEGs by comparing in vitro C2C12 cells and omics data for human samples. The samples were grouped according to 4‐HC treatment status and age (younger versus older), with pairwise comparisons made using EdgeR (version 3.36.0).^[^ ^52^ ^]^ We applied a relatively permissive criterion (false discovery rate <0.05, log(fold change [FC]) > |0.2|) to account for species heterogeneity. The Supporting Methods describe the functional enrichment analysis of GO terms among gene clusters. Additionally, we predicted protein–protein interactions using the Search Tool for Retrieval of Interacting Genes/Proteins (STRING) database.^[^ ^14^ ^]^

##### Experimental Animals

The Animal Ethics Committee under the Institutional Animal Care and Use Committee of National Taiwan University approved the research protocol (Approval no.: 20 190 229). The protocol was developed in compliance with the Guide for the Care and Use of Laboratory Animals from the National Research Council, and all experiments complied with the Animal Research: Reporting of In Vivo Experiments guidelines. We used male C57/BL6J mice (aged 52–54 weeks) provided by the Taiwan National Laboratory Animal Center in Taipei.^[^ ^33^ ^]^ Mice were maintained at a temperature of 24 ± 2 C with a 12‐h light/dark cycle. Relative humidity was maintained at 55 ± 15% throughout the experiment. The animals were acclimatized over one week, which was followed by a 12‐week intervention period. During this period, the mice were fed standard chow, specifically, LabDiet 5010 chow (LabDiet, St. Louis, MO, USA). Animals were monitored for any potential symptoms of disease throughout the experimental period.

##### In Vivo Experimental Design

**Figure 5:**
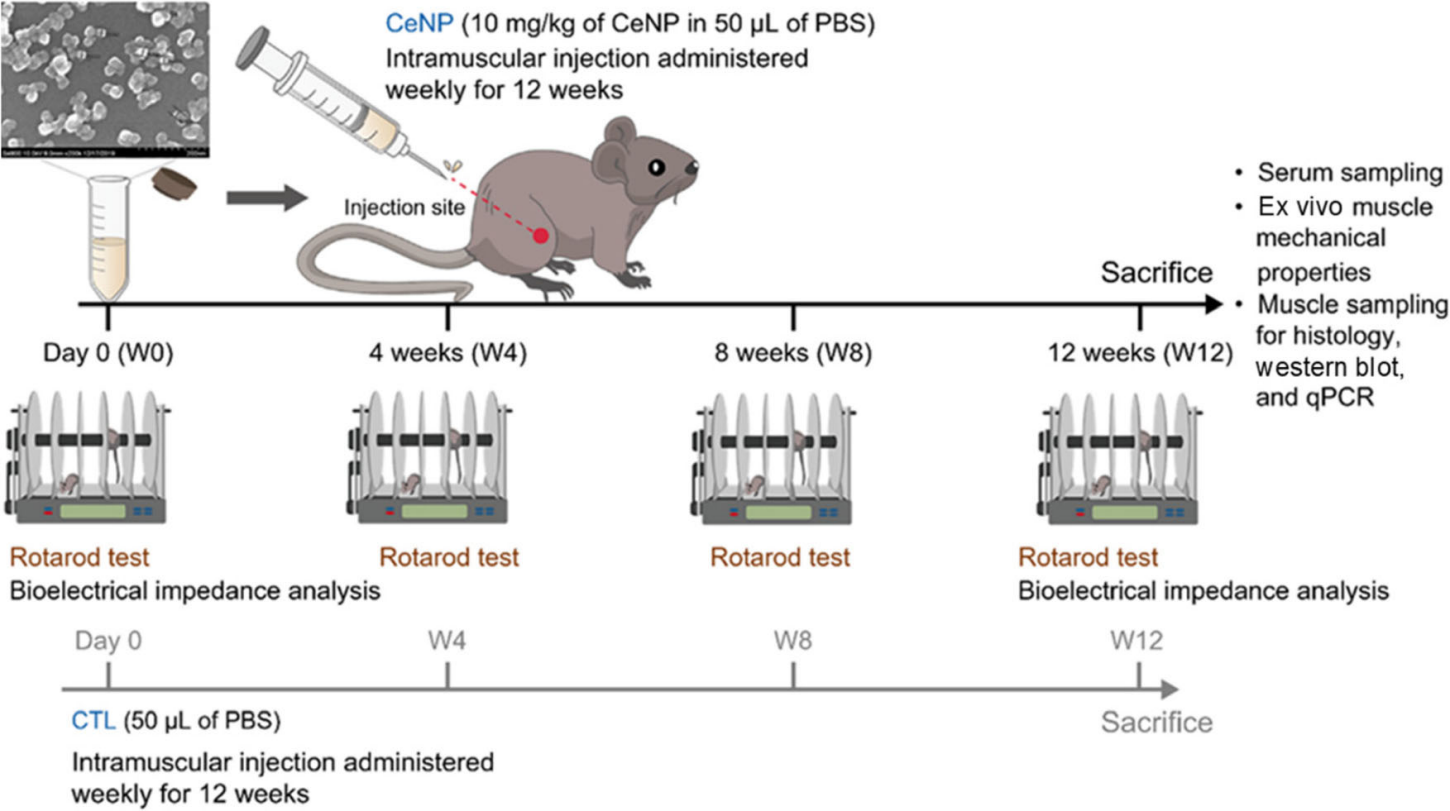
Schematic of the in vivo study. Groups were given weekly intramuscular injections. Control and CeNP groups were administered 50 μL PBS and 10 mg/kg CeNPs in 50 μL PBS, respectively.

According to previous studies, the intraperitoneal injection of CeNPs at a concentration of 10 mg/kg body weight effectively suppresses oxidative stress, inflammation, renal fibrosis, and apoptosis in the kidney, and ameliorates the deterioration of kidney function in a murine model of chronic kidney disease.^[^ ^29^ ^]^ Furthermore, the intravenous injection of CeNPs at a concentration of 10 mg/kg body weight effectively protects the kidney against renal ischemia–reperfusion injury and renal fibrosis in a murine model of renal ischemia–reperfusion injury.^[^ ^30^ ^]^ Therefore, we applied a dose of 10 mg/kg CeNPs in this study. **Figure** **5** illustrates the experimental timeline and describes the animal groups receiving weekly intramuscular injections of the following: 50 μL PBS (control group; n = 5) and 10 mg/kg CeNPs in 50 μL PBS (CeNP group; n = 5). The injections began at 53–54 weeks of age. The experiment was conducted for 12 weeks, with the injections administered in the right quadriceps starting from week 0. The mice were euthanized at 65–66 weeks of age. The motor performance was assessed using the Rotarod test (Ugo Basile Srlmotor function unit, 47 600–Mouse Rota‐Rod; Comerio, VA, Italy). The test involved accelerating the rod (3.2 cm in diameter) from 2 rpm to 40 rpm over 300 s. This test was performed every four weeks to assess changes in the motor performance of mice. After the test, the mice were returned to their cages with access to food and water for 10 min. A total of six trials were conducted, with the first three trials serving as training data and subsequent trials used to collect measurements of the time taken to fall (s); the means of these measurements were used in subsequent analyses. Bioelectrical impedance analysis, which included phase angle calculation (described in the Supporting Information), was conducted at weeks 0 and 12. After the mice were euthanized, we obtained muscle and serum samples to identify any differences between the control and CeNP groups. Profiling of the ex vivo muscle mechanical properties, the Quantibody array (cat. no. QAM‐CAA‐4000; RayBiotech Life, Norcross, GA, USA), and blood biochemistry assays were conducted as described in the Supporting Information. Protein and gene expression profiling was conducted using the same methods as those used in the in vitro experiment, with α‐tubulin (GTX112141; GeneTex, Irvine, CA, USA; 55 kDa) and glyceraldehyde‐3‐phosphate dehydrogenase (GAPDH) used as the internal controls.

##### Histology

Skeletal muscle tissues from the quadriceps were fixed in 10% formalin, left overnight (16 h) at 24 ± 2 C, then dehydrated and embedded in paraffin. We sectioned the tissue blocks to a thickness of 4 μm from the thickest middle part of the muscle,^[^ ^53^ ^]^ then assessed the CSA (μm^2^) of the myofibers by hematoxylin and eosin staining. We soaked the deparaffinized slides first in hematoxylin (Glostrup, Denmark) then in eosin Y solution at 24 ± 2 °C (Sigma‐Aldrich). Subsequently, we viewed the slides with an Olympus optical microscope (Nagano, Japan). ImageJ software was used to estimate the mean CSA values. A minimum of 100 fibers were counted per muscle, from which the mean CSA was calculated.^[^ ^53^ ^]^ Data were represented as the mean ± standard deviation (SD), with three samples analyzed per group. The hindlimb muscles and liver tissues were also removed and fixed in 2.5% phosphate‐buffered glutaraldehyde (0.1 M, pH 7.4) for 24 h, postfixed with 1% osmium tetroxide for 2 h, desiccated in a graded alcohol series, cleared with toluene, and polymerized with EMbed‐812, Electron Microscopy Sciences, Hatfield, PA, USA (EPON). Ultrathin sections (70–90 nm) prepared by ultramicrotomy were contrasted with uranyl acetate and lead citrate, and examined under TEM (JEM 2010 F Microscope, JEOL Ltd., Tokyo, Japan).

##### SiRNA Transfection

For knockdown of CILP2, we transfected C2C12 cells with Lipofectamine 2000 obtained from Invitrogen (Carlsbad, CA, USA) according to the manufacturer's instructions.^[^ ^14^ ^]^ We used siGENOME Mouse CILP2, which is designed to target CILP2 (GenBank NM_026818) and comprises an siRNA‐SMARTpool (SMARTpool, Dharmacon, Lafayette, CO, USA) selected using a multi‐component algorithm, for effective and specific silencing. Each SMARTpool reagent combines four SMART selection‐designed siRNAs into a single pool. The sequences of the siGENOME Mouse CILP2 siRNAs were as follows: CAUCAUCCUUGAAGAGUUA, GCGAACCUAUGGCAUGUUU, GCGAAAGGGUGCACGCCAA, and GGCGACAUCCGUAGGGAGA. C2C12 cells were seeded into a plate with 96 wells at a cell seeding density of 10 000 cells per well. After this seeding process, the cells were incubated for 24 h until they reached full adhesion. Subsequently, the specific siRNA‐SMARTpool (SMARTpool, Dharmacon, Lafayette, CO, USA) was transfected, 30 μM 4‐HC was added, and cells were further incubated for 4 h.

##### Statistical Analyzes

We obtained data from three separate experiments, with values presented as the mean ± SD. The Kolmogorov–Smirnov test was applied to assess data normality. We executed two‐group comparisons by employing unpaired two‐tailed *t*‐tests or two‐way repeated measurement analysis of variance (ANOVA) with Dunnett's post‐hoc test. We executed three‐group comparisons by employing one‐way ANOVA with Bonferroni correction. For power analyzes, we used G*Power (version 3) software, developed by Universität Düsseldorf (Germany), with two‐way repeated measurement ANOVA, where *α* = 0.05 was considered to indicate two‐sided significance. We determined the effect size with reference to previous studies involving behavioral tests in sarcopenic mice.^[^ ^10^ ^]^ With five mice included in each treatment group, *α* = 0.05, and a 15% performance difference between treatment groups (partial η^2^ = 0.33), the statistical power exceeded 80% according to the Rotarod test. We referenced previous oxidative stress and chronic inflammation studies to determine the effect size both in vitro and in vivo.^[^ ^14^ ^]^ For a sample size of three per treatment group, we calculated the statistical power at *α* = 0.05. We performed all statistical analyzes using GraphPad Prism version 5 (GraphPad Software, La Jolla, CA, USA) and SPSS version 20 software (IBM, Armonk, NY, USA). We considered findings with two‐tailed *p* values of <0.05 to be statistically significant.

## Conflict of Interest

The authors declare no conflict of interest.

## Author Contributions

**Hui‐Min David Wang**: conceptualization; formal analysis; funding acquisition; investigation; methodology development; project administration; laboratory resources; statistical software; supervision; validation; writing—original draft; writing—review and editing. **Yu‐Ling Yu**: conceptualization; data curation; formal analysis; methodology development; visualization; writing—original draft. **Ya‐Jyun Liang**: conceptualization; data curation; formal analysis; investigation; methodology development; validation; visualization. **Chia‐Yih Wang**: conceptualization; formal analysis; methodology development; resources. **Feng‐Huei Lin**: conceptualization; formal analysis; funding acquisition; investigation; methodology development; project administration; laboratory resources; statistical software; supervision; validation; writing—original draft; writing—review and editing. **Wei‐Chih Lien**: conceptualization; data curation; formal analysis; funding acquisition; investigation; methodology development; project administration; laboratory resources; validation; visualization; writing—original draft; writing—review and editing. **Yang‐Chen Lin**: data curation; formal analysis; investigation; validation. **Huei‐Cih Chang**: data curation; formal analysis; investigation; validation.

## Ethics Approval Statement

The Animal Ethics Committee under the Institutional Animal Care and Use Committee of National Taiwan University approved the research protocol (Approval no. 20 190 229). The protocol was developed in compliance with the Guide for the Care and Use of Laboratory Animals from the National Research Council, and all experiments complied with the Animal Research: Reporting of In Vivo Experiments guidelines.

## Supporting information

Supplementary Material

## Data Availability

The data supporting the study results can be found at GTEx Portal (https://gtexportal.org/home/, accessed on August 2, 2021). We obtained the data for the analyzes conducted in this study from https://gtexportal.org/home/datasets (accessed on August 2, 2021). We downloaded skeletal muscle RNA sequencing data (n = 298; GTEx_Analysis_2017‐06‐05_v8_RNASeQCv1.1.9_gene_reads.gct.gz) in addition to deidentified samples and subject annotations (GTEx_Analysis_v8_Annotations_SampleAttributesDS.txt, GTEx_Analysis_v8_Annotations_SubjectPhenotypesDS.txt) from the GTEx Portal (https://gtexportal.org/home/datasets). In addition, we downloaded the median transcripts per million for every tissue from the GTEx Portal (GTEx_Analysis_2017‐06‐05_v8_RNASeQCv1.1.9_gene_median_tpm.gct.gz).
