# Supplementary material for: Cerium Oxide Nanoparticles Achieve Long‐Lasting Senescence Inhibition in an Aging Mouse Model of Sarcopenia via Reactive Oxygen Species Scavenging and CILP2 Downregulation
Source: Small Sci. 2025 Jun 26;5(8):2500208. doi: 10.1002/smsc.202500208 (PMC12362745; doi:10.1002/smsc.202500208)
Supplement: Supplementary file 1 — Supplementary Material [file SMSC-5-2500208-s001.pdf]

Supporting Information

Cerium Oxide Nanoparticles Achieve Long-lasting Senescence Inhibition in an Aging Mouse Model of Sarcopenia via Reactive Oxygen Species Scavenging and CILP2 Downregulation

Wei-Chih Lien, Yu-Lin Yu, Ya-Jyun Liang, Chia-Yih Wang, Yang-Chen Lin, Huei-Cih Chang, Feng-Huei Lin, Hui-Min David Wang**

Supporting Methods

Transmission electron microscopy analysis of cerium oxide nanoparticles

The grain size, interplanar spacing, and grain size distribution of cerium oxide nanoparticles (CeNPs) were examined using transmission electron microscopy (JEM 2010F Microscope, JEOL Ltd., Tokyo, Japan). CeNPs were dispersed in 95% ethanol with 10 min of ultrasonication. Subsequently, 2–10 μL of the solution was deposited on a copper grid and air-dried. The interplanar spacing and selected area electron diffraction patterns of crystals were measured and precisely analyzed using DigitalMicrograph (version 3; Gatan, Pleasanton, CA, USA).

Hydrodynamic size assessment of CeNPs

The hydrodynamic size of the CeNPs was determined using a Zetasizer NanoZS analyzer from Malvern Instruments (Worcestershire, UK) with dynamic light scattering. The intensity of the scattered light was detected at 90° relative to the incident beam. To verify the dispersion of synthesized CeNPs in various solvents, the CeNPs were dispersed in water that

had been twice distilled, ultrasonicated for 10 min, and evaluated at 25 °C (n = 3). We analyzed the data using Zetasizer (version 6.20; Malvern Instruments).

X-ray diffraction analysis of CeNPs

An X-ray diffractometer was used to thoroughly examine the lattice structures of synthesized CeNPs for X-ray diffraction (XRD) analysis. As described in the Experimental Section, we centrifuged the CeNP solution for 30 min at 9961 ×*g*, then discarded the supernatant to isolate the CeNP precipitate. We then oven-dried the precipitate at 60 °C for more than 8 h, ground it into a powder, and performed comprehensive XRD analysis. The phases of the CeNPs were characterized using TTRAX III (Rigaku, Danvers, MA, USA) equipped with a rotation anode with Cu Kα radiation (λ = 1.542 Å) at 40 kV and 20 mA. The scan rate was 0.025°/step at 5 s/step, and the two-theta range was 10–80°. We analyzed the data using MDI JADE (version 6.5) software developed by Materials Data (Liverpool, CA, USA). The Scherrer equation was used to calculate the average diameter from the XRD data.

Calculation of specific surface area of CeNPs

We used the gas adsorption method to determine the specific surface area with a surface analyzer (ASAP2020; Micromeritics, Norcross, GA, USA). CeNP samples were prepared in the same manner as that for XRD analysis. Dried CeNP powder was degassed at room temperature (24 ± 2 °C); the temperature and humidity were set to 25 °C and 55%, respectively.

Surface characterization of CeNPs

Surface characterization of CeNPs was performed using X-ray photoelectron spectroscopy with a Theta Probe from Thermo Fisher Scientific (Waltham, MA, USA); an Al Kα source

was used to analyze the oxidation state of Ce and evaluate the Ce^{4+}/Ce^{3+} ratio. The data were processed for peak fitting using Origin software (version 2025).

Antioxidant activity of CeNPs determined by methylene blue assay

Hydrogen peroxide (H_2O_2), hydrochloric acid (HCl), and methylene blue were obtained from Sigma-Aldrich (St. Louis, MO, USA). Methylene blue is an aromatic and heterocyclic dye with a molecular formula of $C_{16}H_{18}CIN_3S$ and a maximum absorption wavelength of 662 nm in the oxidative state.[1] The mixture was prepared by combining 0.375 mL of a 10 mg/L methylene blue solution, 0.15 mL of a 1 M HCl solution, and 0.15 mL of a 1 M H_2O_2 solution. For the CeNP experimental group, we added 0.1 mL of 0.1 μg/mL CeNPs and diluted with water until the final volume reached 1.5 mL. For the control group, we diluted the mixture directly with water to achieve a final volume of 1.5 mL. The final solution had a pH of 1, with a methylene blue concentration of 2.5 mg/L and a H_2O_2 concentration of 0.1 M. In the CeNP group, the CeNP concentration was 6.67 ng/mL. The reaction progress was monitored using UV-Vis spectroscopy (JASCO V-670, Tokyo, Japan) at 662 nm, with measurements taken at the baseline and at 1, 2, and 4 min.[1]

Assay for reactive oxygen species (ROS) detection in cells

We used the 2′,7′-dichlorofluorescein diacetate (DCFDA) cellular ROS detection assay (ab113851; Abcam, USA) to evaluate intracellular ROS levels. We seeded C2C12 cells in 96-well tissue culture plates at a density of 10 000 cells per well and incubated the cells for 24 h to achieve full cell adhesion. For the ROS detection assay, we added 4-HC solution to the culture medium at a final concentration of 30 μM. The sample was left to stand for 4 h before determining the intracellular ROS concentration using the SpectraMax i3x multimode microplate reader (Molecular Devices, USA) or a flow cytometer. The images were captured using a fluorescence microscope (IX51; Olympus, Japan).

To test the ability of CeNPs to reduce the production of intracellular ROS induced by 4-HC, we added 10 μg/mL CeNP to the medium and cultured the cells for 24 h before 4 h of treatment with 4-HC. We washed the cells with PBS and incubated them with 25 μM DCFDA reagent for 45 min. Then, we measured the fluorescence intensity of C2C12 cells treated with different concentrations of CeNPs according to Equation (1) in the Experimental Section for dichlorofluorescein fluorescence (%).

For flow cytometry, cells were washed twice with PBS and suspended in 1 mL trypsin. Individual cells were suspended by gentle pipetting up and down three times before flow cytometry analyses. Signals were detected using a 525 nm bandpass filter (FITC) on a CytoFLEX flow cytometer (Beckman Coulter, Brea, CA, USA), and data were analyzed using Kaluza 2.3 software (Beckman Coulter), where a shift to the right indicates increased ROS levels.

RNA sequencing

We extracted total RNA from C2C12 cells exposed to various treatment conditions (n = 3 in each group) and established sequencing libraries using the TruSeq stranded mRNA library prep kit from Illumina (RS-122-2101; San Diego, CA, USA). The libraries were sequenced on an Illumina NovaSeq 6000 platform, and the data were analyzed with EBSeq (version 1.16.0) to identify differentially expressed genes (DEGs), as described elsewhere.[2]

Gene Ontology enrichment analysis

We conducted Gene Ontology (GO) enrichment analysis using the Database for Annotation, Visualization, and Integrated Discovery (version 6.7) software package.[3] The R package clusterProfiler (version 4.0.0) was used to conduct functional enrichment analysis for GO terms among the gene clusters, with DEGs[4] used to identify the 20 GO terms that were most significantly enriched, as described previously.[2]

Bioelectrical impedance analysis

The mice were subjected to bioelectrical impedance analysis measurements to evaluate electrical conductivity in the muscles. The resistance and phase angle were calculated as described previously, with 27-G needles used as electrodes inserted at specific points on the bodies of the mice.[5, 6] Bioelectrical impedance analysis can be used to indirectly measure body composition, including fat-free and body cell mass, and calculate the phase angle according to resistance and reactance. Such analyses indicate muscle quality, integrity, and functionality and cell membrane permeability.[7, 8] The measurements were conducted at weeks 0 and 12, with three consecutive measurements taken at the same electrode position.[6]

Ex vivo muscle mechanical properties

The passive properties of the muscles (tensile strength in Newtons [N]) were measured on isolated muscles using tensile tests. We placed a 250-N spring clamp on the upper and lower ends of the calf muscle (approximately at the knee and ankle, respectively). The calf muscle was stretched using a static tensile testing machine, namely, the MTS Criterion 42.503 Test System (Eden Prairie, MN, USA), at 20 mm/min to assess the maximum tensile strength during elongation at failure.[9]

Quantibody array profiling and blood biochemistry assays

Blood was collected from six C57BL/6J mice exposed to control and treatment conditions. Samples were analyzed using the Quantibody array platform to assess multiple protein profiles, as previously described.[2] The levels of different pro-inflammatory cytokines in the control and CeNP groups were compared. Blood biochemistry assays were performed on mouse serum using the Roche Cobas c111 analyzer (Roche Diagnostics, Indianapolis, IN,

USA). Levels of creatine kinase, lactate dehydrogenase, alanine aminotransferase, and creatinine were compared between the control and CeNP groups.

Supporting References

[1] M. A. Pugachevskii, D. S. Rasseko, V. A. Stupin, N. E. Manturova, E. B. Artyushkova, E. V. Silina, *J. Mol. Liquid*. **2024**, *404*, 124946.

[2] W. C. Lien, X. R. Zhou, Y. J. Liang, C. T. S. Ching, C. Y. Wang, F. I. Lu, H. C. Chang, F. H. Lin, H. D. Wang, *Bioeng. Transl. Med.* **2023**, *8*, e10346. https://doi.org/10.1002/btm2.10346.

[3] B. T. Sherman, M. Hao, J. Qiu, X. Jiao, M. W. Baseler, H. C. Lane, T. Imamichi, W. Chang, *Nucleic Acids Res.* **2022**, *50*, W216. https://doi.org/10.1093/nar/gkac194.

[4] T. Wu, E. Hu, S. Xu, M. Chen, P. Guo, Z. Dai, T. Feng, L. Zhou, W. Tang, L. Zhan, X. Fu, S. Liu, X. Bo, G. Yu, *Innovation (Camb).* **2021**, *2*, 100141. https://doi.org/10.1016/j.xinn.2021.100141.

[5] J. Li, W. L. Staats, A. Spieker, M. Sung, S. B. Rutkove, *PLoS One* **2012**, *7*, e45004. https://doi.org/10.1371/journal.pone.0045004.

[6] D. Smith, M. Johnson, T. Nagy, *Int. J. Body Compos. Res.* **2009**, *7*, 21.

[7] L. C. Ward, S. Brantlov, *Rev. Endocr. Metab. Disord.* **2023**, *24*, 381. https://doi.org/10.1007/s11154-022-09780-3.

[8] Y. Akamatsu, T. Kusakabe, H. Arai, Y. Yamamoto, K. Nakao, K. Ikeue, Y. Ishihara, T. Tagami, A. Yasoda, K. Ishii, N. Satoh-Asahara, *J. Cachexia Sarcopenia Muscle* **2022**, *13*, 180. https://doi.org/10.1002/jcsm.12860.

[9] T. Mazurek, M. Strankowski, M. Ceynowa, M. Rocławski, *Clin. Biomech. (Bristol, Avon).* **2011**, *26*, 415. https://doi.org/10.1016/j.clinbiomech.2010.11.014.

Supporting Tables

Table S1. Serum biochemical analysis (mean [standard deviation], n = 5 each).

	**Control**	**CeNP**
CK (U/L)	1054.4 (647.4)	958.4 (544.2)
LDH (U/L)	3101.4 (737.3)	2785.6 (966.3)
ALT (U/L)	46.4 (4.0)	42.4 (7.8)
Crea (mg/dL)	0.2 (0.0)	0.2 (0.0)

CeNP: cerium oxide nanoparticle; CK: creatine kinase; LDH: lactate dehydrogenase; ALT: alanine aminotransferase; Crea: creatinine.

Table S2. Primers used for quantitative polymerase chain reaction

Mouse primers	Sequence (5′–3′)
α Tubulin forward	ACACCTTCTTCAGTGAGACAGG
α Tubulin reverse	CTCATTGTCTACCATGAAGGCAC
Serpine1 forward	CCTCTTCCACAAGTCTGATGGC
Serpine1 reverse	GCAGTTCCACAACGTCATACTCG
Cxcl10 forward	ATCATCCCTGCGAGCCTATCCT
Cxcl10 reverse	GACCTTTTTTGGCTAAACGCTTTC
Il6 forward	ACCCCAATTTCCAATGCTCTCC
Il6 reverse	AACGCACTAGGTTTGCCGAG
Tnfα forward	GGTGCCTATGTCTCAGCCTCTT
Tnfα reverse	GCCATAGAACTGATGAGAGGGAG
Atrogin-1 forward	CTTCTCGACTGCCATCCTGGAT
Atrogin-1 reverse	TCTTTTGGGCGATGCCACTCAG
p21 forward	CAGCATAGAGCAGGACATGGAG
p21 reverse	GAACAGCGGTAGTATCAGCCAG

Supporting Figures

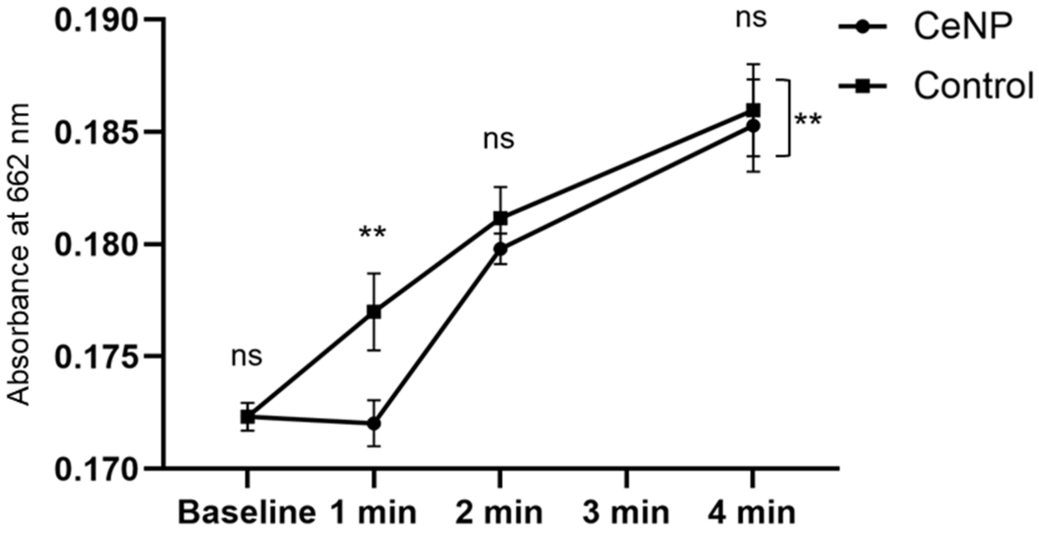

Figure S1. Measured absorbance of methylene blue at a wavelength of 662 nm at the baseline and at 1, 2, and 4 min in the presence of methylene blue (2.5 mg/L), hydrogen peroxide (0.1 M), and with and without CeNPs (6.67 ng/mL). Values are the mean ± standard deviation. Two-group comparisons were conducted via a two-way repeated measurement analysis of variance (ANOVA) with Dunnett's post-hoc test. *p < 0.05; **p < 0.01; ***p < 0.001; ns, not significant. CeNP, cerium oxide nanoparticle.

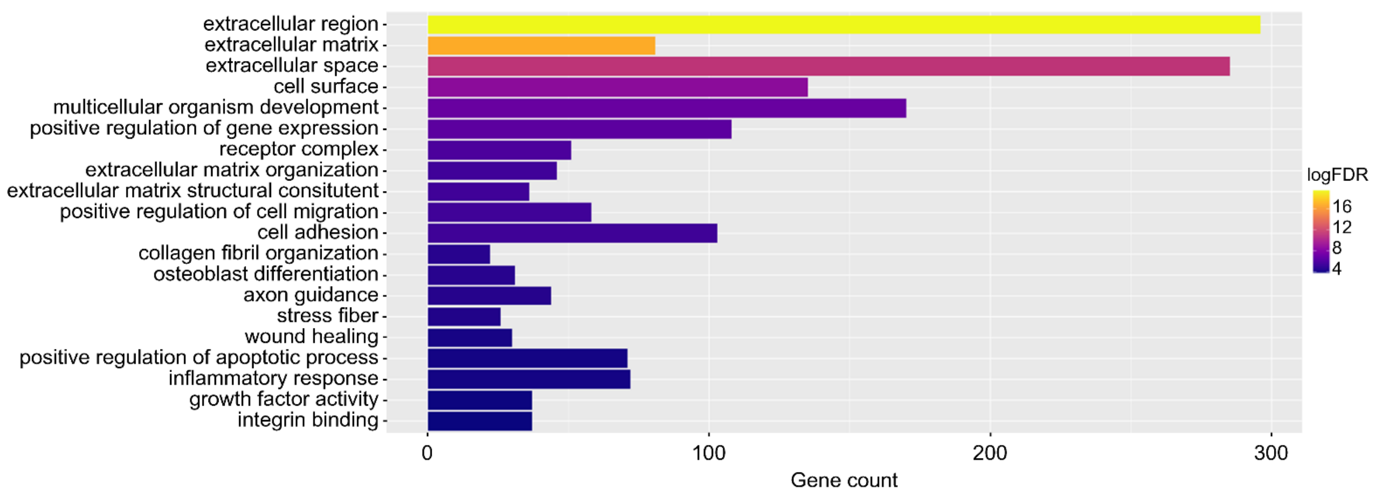

Figure S2. Comparative Gene Ontology enrichment analysis between control and 4-HC-treated C2C12 cells.

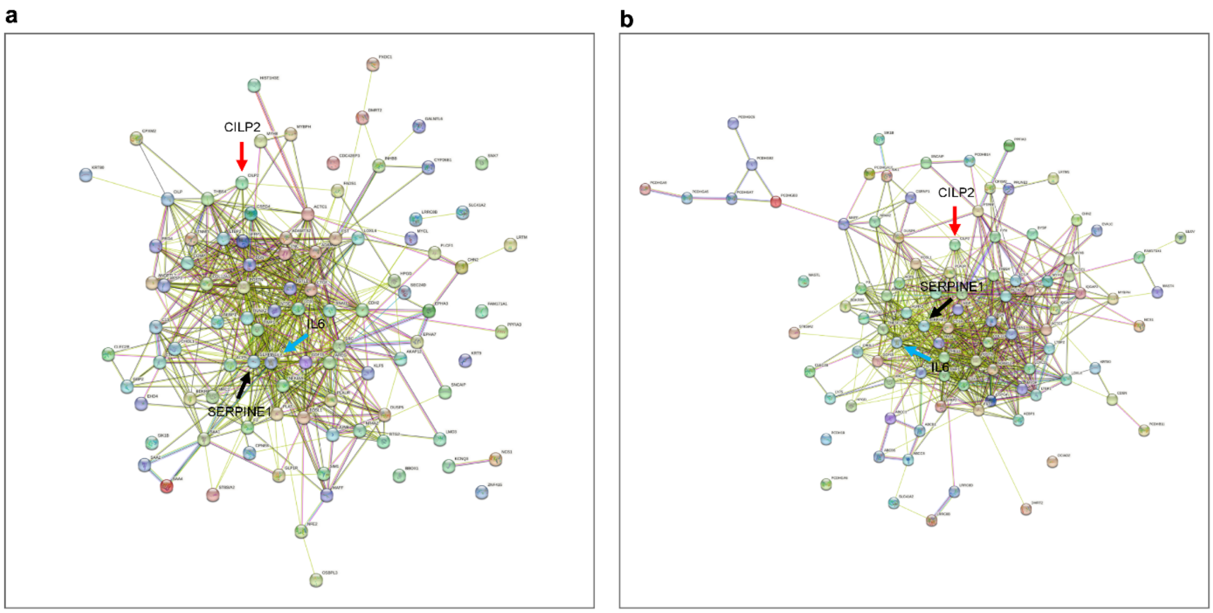

Figure S3. Protein–protein interactions (PPI) in differentially expressed genes (DEGs) identified from the Search Tool for Retrieval of Interacting Genes/Proteins database. Red arrow indicates CILP2, blue arrow indicates IL6, and black arrow indicates SERPINE1. **a**. PPI for DEGs common to 4-HC vs. control in C2C12 cells and human muscle samples. **b**. PPI for DEGs common to 4-HC vs. 4-HC+CeNP in C2C12 cells and human muscle samples.

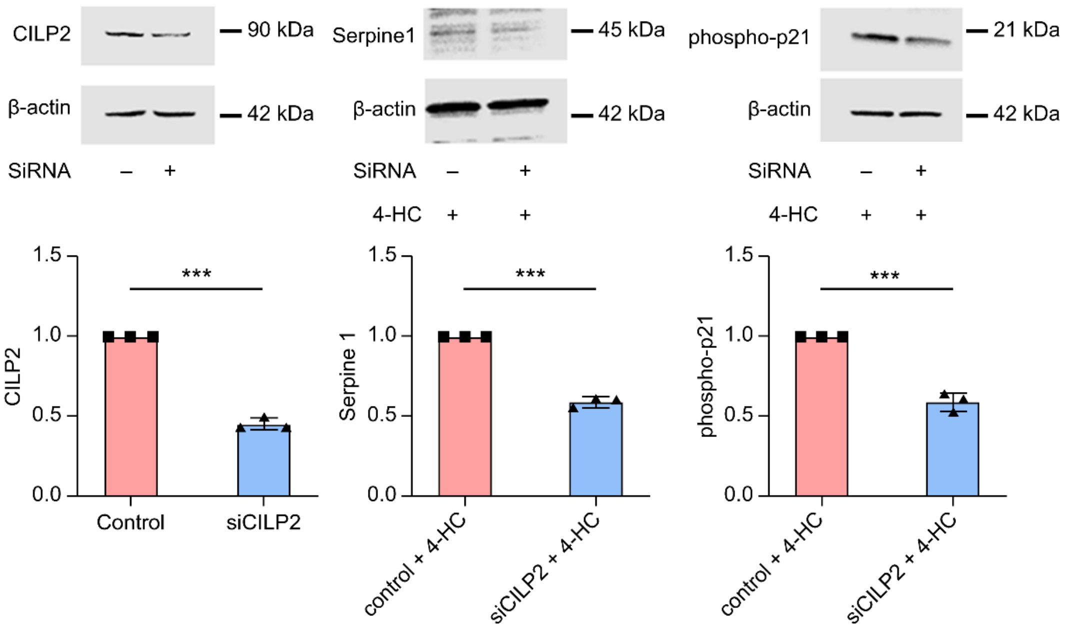

Figure S4. Western blot analysis of siRNA-mediated CILP2 knockdown in C2C12 cells (left), and the expression of SERPINE1 (middle) and phospho-p21 (right) in siCILP2-knockdown C2C12 cells treated with 4-hydroperoxy cyclophosphamide (4-HC). Values are the mean ± standard deviation. Data were compared via unpaired two-tailed *t*-tests. *$p < 0.05$; **$p < 0.01$; ***$p < 0.001$; ns, not significant.

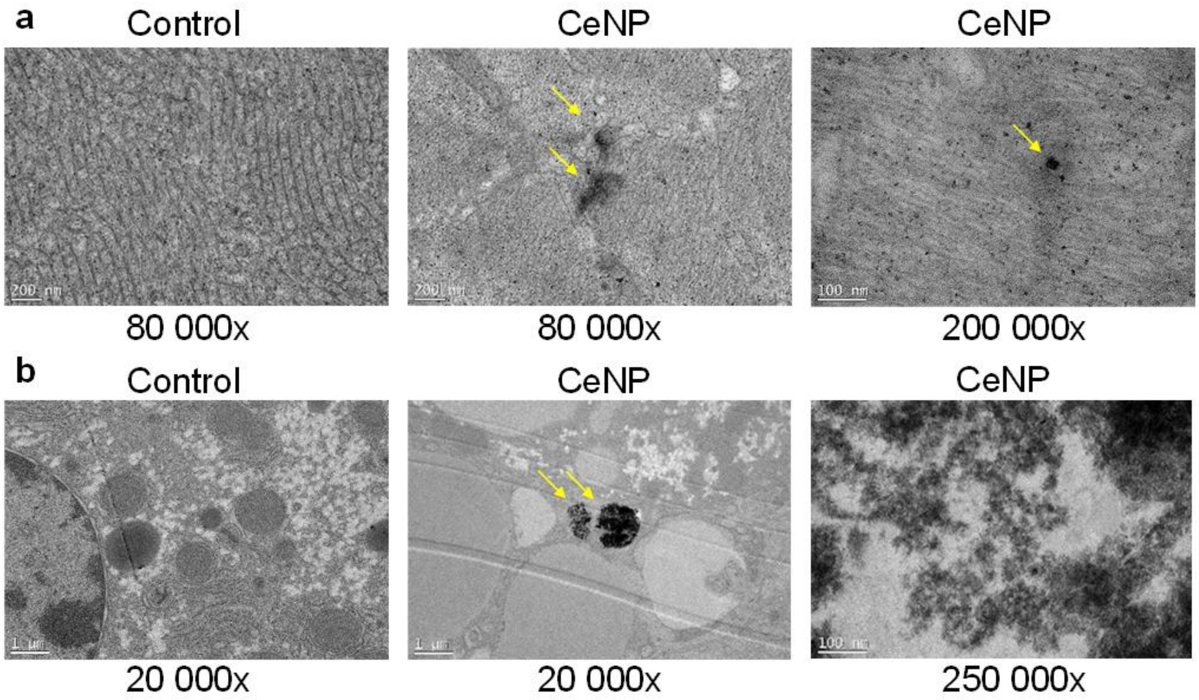

Figure S5. Transmission electron microscopy images of muscle and liver samples from control and CeNP groups. (A) Muscle samples from the control group were observed at 80 000× magnification with an accumulation of parallel tubules. Quadriceps and calf muscle samples from the CeNP group were observed at 80 000× and 200 000× magnification, respectively. Yellow arrows indicate CeNPs in the muscle cytoplasm. (B) Liver samples from the control and CeNP groups were observed at 20 000× magnification. Yellow arrows indicate CeNPs in the liver cytoplasm, with CeNP aggregation observed at 250 000× magnification.
